# An Unexpected Near Term Pregnancy in a Rudimentary Uterine Horn

**DOI:** 10.1155/2013/307828

**Published:** 2013-04-27

**Authors:** Elisabete Gonçalves, João Pedro Prata, Sandra Ferreira, Rita Abreu, Jorge Mesquita, Agostinho Carvalho, Paula Pinheiro

**Affiliations:** Department of Gynecology and Obstetrics, Unidade Local de Saúde do Alto Minho, Estrada de Santa Lúzia, No. 13, 4901-858 Viana do Castelo, Portugal

## Abstract

Unicornuate uterus occurs due to a complete or partial nondevelopment of one Mullerian duct; sometimes it is associated with a rudimentary horn, which can communicate or not with uterine cavity or contain functional endometrium. Pregnancy in a rudimentary horn is rare and the outcome almost always unfavorable, usually ending in rupture during the first or second trimester with significant morbidity and mortality. Despite the availability and advances on imagiologic procedures, recognition of this ectopic pregnancy is frequently made at laparotomy after abdominal pain and collapse. The authors describe a case of a primigravida with 34 weeks of gestation admitted with a preeclampsia with severity criteria. A cesarean for fetal malpresentation was done and, unexpectedly, a rudimentary horn pregnancy was found with a live newborn. In the literature, few reports of a horn pregnancy reaching the viability with a live newborn are described, enhancing the clinical importance of this case. A review of literature concerning the epidemics, clinical presentation, and appropriate management of uterine horn pregnancies is made.

## 1. Introduction

Abnormalities of embryogenesis of Mullerian duct system resulting in congenital anomalies of female genital tract are relatively common [1]. The exact incidence of these anomalies is difficult to determine since usually they are no clinically symptomatic [1]; however, it is estimated to occur in 2 to 4 percent of women with normal reproductive outcomes, and such prevalence could be higher among women with infertility or obstetric complications [1–3].

Unicornuate uterus is a type II Mullerian anomaly according to the American Fertility Society classification system [1] that occurs due to a complete or partial failure of development of one Mullerian duct and incomplete fusion with contralateral side [1–3]. The failed Mullerian duct leads to the formation of an isolated hemiuterus without a contralateral structure (in complete failure) to various degrees of a rudimentary horn (in partial failure) [1–3]. This rudimentary horn is subclassified into communicating or non-communicating with uterine cavity and a horn with no cavity [1–3].

Unicornuate uterus accounts for 5 percent of all Mullerian anomalies, occurring in general population, approximately, to 1 in 4020 women [1, 3]; in about 84 percent of these cases a contralateral rudimentary horn exists, almost always of a non communicating type [4].

Unicornuate uterus is related to an increased risk of infertility, first trimester miscarriage (24.3%), second trimester miscarriage (9.7%), ectopic pregnancy (2.7%), preterm labor (20.1%), intrauterine growth restriction, intrauterine fetal demise (10.5%), placenta, accreta and fetal malpresentation [1–3]. Renal abnormalities coexist up to 40 percent of cases of unicornuate uterus [1, 3]. Other associated anomalies such as an ectopic ovary tissue and, more rarely, absent ipsilateral gonad could occur [1, 3].

The presence of a rudimentary uterine horn with cavity leads to well characteristic gynecologic and obstetrical complications [3]. Most rudimentary horns are asymptomatic; however, some contain functional endometrium, although not necessarily normal [1]. Cyclic or chronic pelvic pain (usually the presenting symptom), hematometra, and endometriosis are often associated in these cases. Besides, the uterine horn could represent a site for ectopic pregnancy, where natural course is rupture during second trimester, with a potentially life-threatening heavy bleeding [2].

Pregnancy in such a rudimentary horn is extremely rare, 10-fold less common than an abdominal pregnancy. We describe an unexpected horn pregnancy reaching the viability with a live newborn, an unusual presentation.

## 2. Case Presentation

A 22-year-old woman, primigravida, presented at our emergency department complaining about diminished fetal movements at 34 weeks of gestation. She had no relevant medical past. Antenatal surveillance was performed at primary health care, and it was uneventful.

On examination, she had oedema of inferior lower limbs, a blood pressure of 156/92 mm Hg, a pulse of 60 beats per minute, and 3+ proteinuria on a dipstick. On gynecological exploration she had normal external genitals, vagina, and cervix appeared macroscopically normal; the cervix was posterior, large with cervical os closed at palpation. Obstetric ultrasound revealed a fetus with present corporal movements, a breech presentation, an estimated fetal weight of 2100 g, normal amniotic fluid, normal inserted placenta, and normal uterine artery Doppler. She was admitted for evaluation of pregnancy-associated hypertension, and corticotherapy for fetal pulmonary maturity was instituted. Monitoring showed a low maternal urinary output, and analytics showed a hemoglobin of 15, 2 g/dL, normal platelet count and liver enzymes, high uric acid (8,5 g/dl), and a 24 h proteinuria of 7,6 g/dL compatible with a severe preeclampsia. Delivery was performed 2 days after admission by cesarean for fetal malpresentation.

At cesarean, a live 2010 g female newborn was extracted from an unusual saccular structure that presented at supra-pubic space. The newborn was transferred to the Neonatology Unity. After placenta delivery and involution, abdomino-pelvic cavity was explored and, unexpectedly, it was found an unicornuate uterus behind and at the left direction of the saccular structure. With such findings, an hysterometry was done, it was understood that the cervix communicated with the left unicornuate uterus, and it was realized that the saccular structure corresponded to a rudimentary horn pregnancy. The horn was connected to the istmic right wall of the uterus by a thin fibromuscular tissue; the unicornuate uterus had a left fallopian tube and a cervix that communicated with vagina; bilateral adnexae were normal. The rudimentary horn was removed. Right ureter was not found.

**Figure 1:**
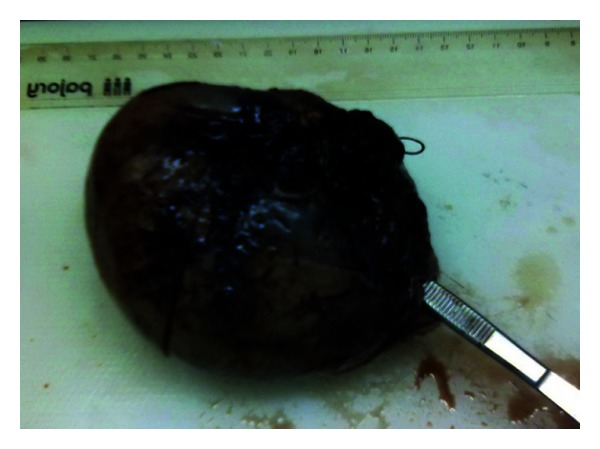
Rudimentary uterine horn after delivery and excision.

**Figure 2:**
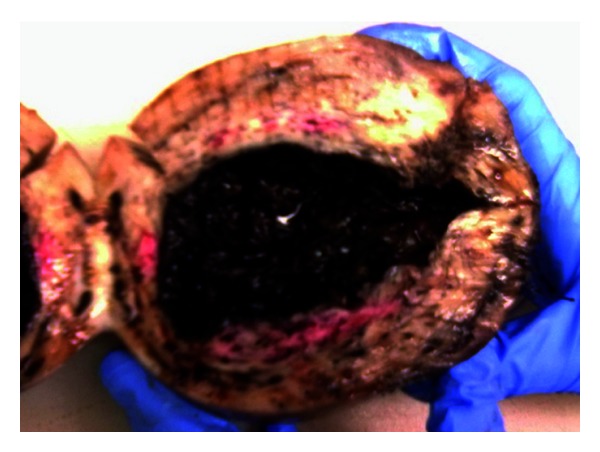
Transversal section of the rudimentary uterine horn, showing a cavity coated with decidualized endometrium.

The postoperative course was favorable with resolution of preeclampsia clinic, and she was discharged with the newborn on the 6th postoperative day. Four weeks later, she was reevaluated for investigation for preeclampsia and to rule out renal abnormalities. She was clinically well. Analytics were normal. MRI was required and showed an unicornuate uterus, normal ovaries, and an absent right kidney with a left vicarious kidney. Pathological evaluation of the specimen confirmed a uterine horn measuring 12 × 10 × 10 cm coated with decidualized endometrium, non communicating type (Figures 1 and 2).

## 3. Discussion

Pregnancy in a non communicating rudimentary horn is uncommon, estimated to occur in 1 per 100000 to 140000 pregnancies [2]. The first described pregnancy in a rudimentary uterine horn was made in 1669 by Mauriceau [5] and, worldwide, it have been described up to now in about 700 cases [6]. Besides, it is rare for such pregnancy to result in a viable fetus: only 10 percent reach term, and the newborn survival rate is about 2% [2, 5] enhancing the importance of this case report.

It is postulated that pregnancy in a non communicating rudimentary horn only could occur due to transperitoneal migration of the spermatozoon or the transperitoneal migration of the fertilized ovum through contralateral tube [3–5, 7].

The natural course of a rudimentary horn pregnancy is rupture during the first or mid-second trimester [1–4, 6]. In the majority of cases, horn rupture occurs before 20 weeks [8]; reports of rupture varying from 5 to 37 weeks are described [5, 8], depending on the horn musculature, variable thickness and distensibility of myometrium [2, 5, 8]. Being the uterine wall thicker and more vascular, bleeding is more severe in rudimentary horn pregnancy rupture, therefore, its common manifestation is an acute abdominal pain with heavy intraperitoneal hemorrahage, that could be life-threatening [2–4, 8]. Nowadays, maternal mortality rate is estimated to be less than 0.5 percent [2, 5], however, in the 19th century it was reported to be around 47 percent [8] and it is related with exsanguination due to horn rupture [9].

Because of reduced expansibility, relatively small volume and anomalous vasculature supplying the rudimentary horn a malformed fetus, fetal growth restriction, oligohydramnios and fetal malpresentation represent other forms of presentation of this condition [3, 9]. The endometrium of the rudimentary horn has been described as thinner and sometimes dysfunctional leading to pathologic placentation, being placenta accreta described with this condition [7]. Failed termination of pregnancy by medical method and uterine evacuation has been reported in obstructive Mullerian anomalies [2, 10].

Early diagnosis of the rudimentary horn pregnancy is essential, but it can be challenging, usually made after laparotomy for acute abdomen [2, 5, 8]. An early bimanual palpation showing a deviated uterus with a palpable adnexal mass, a mass extending outside the uterine angle (Baart de la faille's sign) or displacement of fundus contralateral side with rotation of uterus and elevation of affected horn known as Ruge Simnn Syndrome should lead to a suspicion of a Mullerian anomaly [11].

The availability and advances in ultrasound and magnetic resonance imaging ameliorate the diagnosis of rudimentary horn pregnancy principally at an early gestational age. However, as the gestational age increases, the enlarged pregnant horn can occult adjacent anatomic structures difficuting the diagnosis [4, 8]. The sensitivity of ultrasound to diagnose a pregnant uterine horn could be as low as 30 percent [4]. Tsafrir et al. suggested ultrasound criteria for early diagnosis of this condition that include (1) a pseudopattern of asymmetrical bicornuate uterus, (2) absent visual continuity between the cervical canal and the lumen of the pregnant horn, and (3) presence of myometrial tissue surrounding the gestational sac [3, 7, 8]. MRI has a major role for the diagnosis of Mullerian anomalies and should be considered when a pregnant rudimentary horn is suspected [1, 7].

The classic management of a rudimentary horn pregnancy had been laparotomy with excision of the rudimentary horn and ipsilateral salpingectomy in order to prevent rupture, future ectopic pregnancies, and dysmenorrhea [1, 2, 7]. Hysterectomy may be necessary in massive hemorrhage [2]. Recently, several early diagnosed cases have been treated by laparoscopic approach [2, 7, 12]. The surgical principles to remove a pregnant rudimentary horn are similar to nonpregnant state; however, vascular pedicles are prone to hemorrhage [12]. Medical management with methotrexate or fetocide (in a later pregnancy), and posterior pregnancy rudimentary horn excision by laparoscopy is proposed by Cutner et al. with the aim to shrink the horn and allow a less invasive surgery [12]. A case of a rudimentary horn pregnancy successfully managed with methotrexate administration at an early gestational week was reported [8]. Conservative management during pregnancy, until viability is achieved, has been reported in the literature in selected cases with good accessibility to emergent surgery [2, 8].

Pregnancy in a non communicating uterine horn ending with the delivery of a live newborn is a rare event. In our case, the recognition of a rudimentary horn pregnancy was an accidental finding after a cesarean by a breech presentation in a pregnant woman diagnosed with severe preeclampsia for proteinuria. The patient was a primigravida, and she had no relevant gynecologic history, such as pelvic pain or infertility that could indicate presence of Mullerian anomalies. The pregnancy was monitored at primary health care and it was uneventful until the admission at 34 weeks. At our department, there was no suspicion of a Mullerian anomaly at ultrasound. Sensitivity in detecting rudimentary horn uterus is low [4] and even more difficult to perform the diagnosis with a near term pregnancy related with the enlarged pregnant horn. Fetal malpresentation and preeclampsia (attributed to congenital renal anomalies), as our patient presented, have been reported in association with a rudimentary horn pregnancy [1, 9]. However, these conditions are found in normal pregnancies, and it did not raise the suspicion of the diagnosis. Removal of uterine rudimentary horn is mandatory to reduce the future risk of an ectopic pregnancy and the possibility of dysmenorrhea. Ipsilateral adnexa was conserved in our case, since it appeared to be normal; however, some argue that it may be a potential site for ectopic pregnancy. Investigation to rule out urinary anomalies is fundamental in these patients; imaging our patient revealed an absent kidney in the ipsilateral side, which could be associated with combined preeclampsia. Despite the removal of the rudimentary horn pregnancy, the patient should be advised of the increased risk of ectopic pregnancy and the increased risk of preterm labor related with the unicornuate uterus.

An ectopic pregnancy in a rudimentary horn is rare and carries severe maternal-fetal consequences; antenatal diagnosis is challenging, usually performed at emergent surgery. Therefore, increased awareness is recommended to prevent the morbidity, especially in high risk groups: previous history of pelvic pain and infertility, recurrent miscarriages or late miscarriage, preterm labor, fetal malpresentation, fetal growth restriction, abnormal placentation, preeclampsia or failure induction for termination of pregnancy.
